# Mammographic density is a potential predictive marker of pathological response after neoadjuvant chemotherapy in breast cancer

**DOI:** 10.1186/s12885-019-6485-4

**Published:** 2019-12-30

**Authors:** Ida Skarping, Daniel Förnvik, Hanna Sartor, Uffe Heide-Jørgensen, Sophia Zackrisson, Signe Borgquist

**Affiliations:** 1 Division of Oncology and Pathology, Department of Clinical Sciences, Lund University, Skåne University Hospital, Lund, Sweden; 2 Medical Radiation Physics, Department of Translational Medicine, Lund University, Skåne University Hospital, Malmö, Sweden; 3 Diagnostic Radiology, Department of Translational Medicine, Lund University, Skåne University Hospital, Lund and Malmö, Sweden; 4 Department of Clinical Epidemiology, Aarhus University Hospital, Aarhus, Denmark; 5 Department of Oncology, Aarhus University Hospital, Aarhus, Denmark

**Keywords:** Breast cancer, Mammographic density, Neoadjuvant treatment, Pathological complete response

## Abstract

**Background:**

Our aim is to study if mammographic density (MD) prior to neoadjuvant chemotherapy is a predictive factor in accomplishing a pathological complete response (pCR) in neoadjuvant-treated breast cancer patients.

**Methods:**

Data on all neoadjuvant treated breast cancer patients in Southern Sweden (2005–2016) were retrospectively identified, with patient and tumor characteristics retrieved from their medical charts. Diagnostic mammograms were used to evaluate and score MD as categorized by breast composition with the Breast Imaging-Reporting and Data System (BI-RADS) 5th edition. Logistic regression was used in complete cases to assess the odds ratios (OR) for pCR compared to BI-RADS categories (*a* vs *b-d*), adjusting for patient and pre-treatment tumor characteristics.

**Results:**

A total of 302 patients were included in the study population, of which 57 (18.9%) patients accomplished pCR following neoadjuvant chemotherapy. The number of patients in the BI-RADS category *a, b, c*, and *d* were separately 16, 120, 140, and 26, respectively. In comparison to patients with BI-RADS breast composition *a*, patients with denser breasts had a lower OR of accomplishing pCR: BI-RADS *b* 0.32 (95%CI 0.07–0.1.5), BI-RADS *c* 0.30 (95%CI 0.06–1.45), and BI-RADS *d* 0.06 (95%CI 0.01–0.56). These associations were measured with lower point estimates, but wider confidence interval, in premenopausal patients; OR of accomplishing pCR for BI-RADS d in comparison to BI-RADS a: 0.03 (95%CI 0.00–0.76).

**Conclusions:**

The likelihood of accomplishing pCR is indicated to be lower in breast cancer patients with higher MD, which need to be analysed in future studies for improved clinical decision-making regarding neoadjuvant treatment.

## Background

Mammographic density (MD), reflecting the amount of fibroglandular tissue, is a noncontroversial established risk factor for breast cancer (BC) [1, 2]. Studies aiming at elucidating the role of MD in the adjuvant BC setting have shown that a temporal decrease in MD after a primary BC lowers the risk of future contralateral BC [3]. Similarly, BC patients responding with MD reduction during endocrine treatment have improved long-term survival [4]. The assessment of density can be done visually by a radiologist, using the Breast Imaging-Reporting and Data System (BI-RADS) [5] for breast composition categories, or be calculated by one of many computerized methods. MD assessment by radiologists and digital software show good agreement [6, 7], with both qualitative and quantitative methods of measuring MD showing an association between high MD and risk of developing BC [8]. High MD is not only a risk factor for BC, but it reduces the prospect of detecting a BC, since surrounding breast tissue “masks” the malignancy, known as the masking effect [9]. Accordingly, the 5th edition of BI-RADS breast composition categorization aims at better assessment of density by emphasizing this masking effect [10].

Neoadjuvant systemic BC treatment is provided to an increasing number of patients [11]. In general, there is no difference in recurrence free survival or in overall survival for BC patients receiving adjuvant or neoadjuvant chemotherapy (NACT) [12]. The possibility of personalized treatment and then evaluating treatment response in the primary tumor renders NACT the preferred choice for many patients. Accomplished pathological complete response (pCR) after NACT is considered to be a surrogate marker for improved long-term survival [13, 14]. Consequently, it is urgent to predict responders from non-responders for an optimal clinical decision as early as possible, that is prior to initiation of therapy. Predictive biomarkers are thus needed, potentially covering a range of markers, including patient and tumor characteristics, gene and protein expression and different imaging biomarkers. Established predictors of response to NACT include younger age, triple negative phenotype or grade III tumors [15], and high tumor proliferation (Ki67) [16]. Additional biomarkers, such as imaging biomarkers, are needed to give each patient tailored cancer treatment.

A previously published study, investigating the association between pCR after NACT and MD was restricted to use of a binary classification of MD, comprised of low or high density (cutoff at 25% of the breast consisting of radio-dense tissue), which found that low MD is linked to a higher pCR rate [17].

In this study, we aim to investigate the association between the established BI-RADS breast composition categories for breast density at diagnosis and the pCR rate after NACT in a Swedish consecutive cohort of 302 BC patients. We hypothesized MD to be a predictive marker of pCR to NACT.

## Methods

**Fig. 1:**
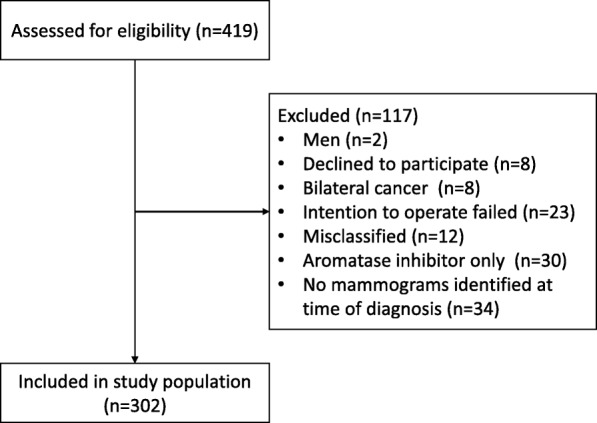
Patient flow chart

All patients receiving any NACT for BC from January 2005 to June 2016 at Skåne University Hospital, Sweden, were identified (*N* = 419). Patients identified as deceased after cross-referencing with the Swedish population registry (*N* = 23), were included without consent. The remainder of the patients were asked for their consent at the time of the study, although a minority (*N* = 8) did not wish to participate. Exclusion criteria were: male gender, did not wish to participate, bilateral BC at time of diagnosis, primary surgery failed, misclassified with respect to neoadjuvant treatment, or there were no mammograms at the time of BC diagnosis. Only patients treated with chemotherapy and/or human epidermal growth factor receptor 2 (HER2)-targeted therapy (trastuzumab) were included. In total, 302 patients were included in the study population (Fig. 1). Menopausal status at time of diagnosis was collected from patient records. Perimenopausal patients (*N* = 13) with irregular menstrual periods (i.e., less than 1 year since the last period) were included in the postmenopausal group. When menopausal status at the time of diagnosis was unknown (*N* = 6), the patient was considered postmenopausal if older than 55, and premenopausal otherwise. A total number of 136 and 166 women were considered premenopausal and postmenopausal, respectively.

Digitally-processed mammograms acquired at the time of diagnosis, prior to systemic cancer treatment, were gathered retrospectively through the Picture Archiving and Communication System as part of the digital medical charts. The mammograms were done at three sites in southern Sweden on different machines: Fujifilm, GE Healthcare, Philips Healthcare, and Siemens Healthineers. Visual assessment and categorization according to the BI-RADS 5th edition was performed by one radiologist (HS), blinded for patient outcomes. Each patient was given a BI-RADS breast composition 5th score from *a* to *d*, with category *a* representing “the breast are almost entirely fatty“, category *b* representing “there are scattered areas of fibroglandular density“, category *c* representing “the breasts are heterogeneously dense, which may obscure small masses“ and category *d*, representing “the breasts are extremely dense, which lowers the sensitivity of mammography“ [5].

Information on tumor size (mm) at diagnosis was estimated with either mammography and/or ultrasound and was retrieved from the radiology report. When both mammography and ultrasound were used for size estimation, a mean value was used in statistical analyses. In a few patients (*N* = 3), only metastases in the axilla, and no tumor in the breast was visualized, and hence not measurable at the time of diagnosis. Additionally, a number of patients (*N* = 10) presented with inflammatory BC at the time of diagnosis and the entire breast was considered cancerous; consequently, no tumor size was assessed prior to treatment, although the MD was estimated from the contralateral non-cancerous breast.

Treatment response was considered as pCR with an absence of any residual invasive cancer in the resected breast specimen and all sampled regional lymph nodes following completion of NACT [18]. Patient and tumor characteristics were retrieved from digital medical charts. Tumors were considered estrogen-receptor (ER) and progesterone-receptor (PR) positive when the respective receptor was stained in more than 10% of the cells, according to Swedish clinical practice. Tumors were considered Ki67 high, when more than 20% of the cells stained positive. Tumors were initially immunohistochemically stained for HER2 and tumors assessed as HER2 2+ or 3+ on immunohistochemical staining, underwent further analyses with fluorescence in situ hybridization (FISH), resulting in either normal or amplified HER2 status. HER2-positivity was defined as either 3+ with an immunohistochemical method and/or amplified with FISH.

The study was approved by the Regional Ethics Committee in Lund, Sweden (#2014/13 and #2016/521).

### Statistical analyses

We summarized baseline data according to BI-RADS breast composition. Categorical variables were summarized by counts and percentages, and continuous variables by their median and interquartile range.

We set up three logistic regression models to estimate odds ratios (OR) and corresponding 95% confidence intervals (CI) for an association between BI-RADS breast composition categories and tumor response. We refer to these models as the crude-, the minimally adjusted-, and the fully adjusted model, respectively. BI-RADS breast composition category *a* was set as a reference for comparison to categories *b*, *c*, and *d*. The crude model included only the BI-RADS breast composition category as an independent variable. In the minimally adjusted model, we adjusted for pretreatment patient characteristics: age, body mass index (BMI), menopausal status, hormone replacement therapy (HRT), and number of pregnancies (categorical: none, 1, 2, 3+). In the fully adjusted model, we included variables from the minimally adjusted model and pre-chemotherapy tumor variables: ER, PR, Ki67, HER2, and tumor size. All three models were restricted to complete cases, i.e., patients without missing values of the variables in the third model. We used generalized estimation equations to account for potential within-hospital correlation, which was done with the REPEATED SUBJECT statement in the GENMOD procedure in SAS.

We repeated the three logistic regression models in subgroups defined by pre−/postmenopausal status. In the subgroup of postmenopausal women, it was not possible to adjust for Ki67 in the fully adjusted model, since there were no patients with a BI-RADS breast composition category *a* among those with Ki67 ≤ 20%. Since none of the postmenopausal women with BI-RADS breast composition *d* accomplished pCR, it was not possible to estimate OR for this category in the logistic regression models for postmenopausal patients.

Statistical analyses were conducted with SPSS (IBM SPSS Statistics for Windows, Version 24.0; IBM Corp Armonk, NY, USA) and SAS (SAS Institute Inc., Version 9.4, Cary, NC, USA). Since this study population is a consecutive cohort, no power estimates were made in advance.

## Results

For the 302 BC patients included in the study (Fig. 1), the distribution of patient characteristics according to MD is presented in Table 1. Patients with very dense breasts (BI-RADS breast composition *d*) were younger, had a lower BMI, were older for their first birth, were more often premenopausal, and were more often current oral contraceptives users than patients with BI-RADS breast composition *a-c*. At the time of diagnosis, tumors in very dense breasts (BI-RADS breast composition *d*) were more likely to be ER-positive, PR-positive, and not highly proliferative (Ki67-score < 20%) than tumors in less dense breasts (Table 2), whereas HER2 status was fairly equally distributed. After NACT, tumors in very dense breasts (BI-RADS breast composition *d*) were more likely to be ER-positive, PR-negative, HER2-negative, not highly proliferative (not a high Ki67-score), and less likely to be cancer-free in the axilla than tumors in non-dense breasts (Additional file 1).

**Table 1:** Patients characteristics according to mammographic density at diagnosis

		BI-RADS^a^ a	BI-RADS b	BI-RADS c	BI-RADS d
Number of patients		16	120	140	26
Age	median (IQR)	59 (54–68)	59 (50–66)	49 (41–60)	44 (37–54)
BMI	median (IQR)	30 (27–35)	27 (24–30)	24 (22–27)	23 (21–26)
Number of pregnancies	0	1 (6.3)	9 (7.5)	25 (17.9)	3 (11.5)
	1		21 (17.5)	22 (15.7)	3 (11.5)
	2	6 (37.5)	34 (28.3)	51 (36.4)	10 (38.5)
	3+	9 (56.3)	55 (45.8)	41 (29.3)	10 (38.5)
	missing		1 (0.8)	1 (0.7)	
Age at first birth	median (IQR)	24 (23–24)	26 (23–29)	29 (25–33)	29 (28–33)
Age at menarche	median (IQR)	13 (13–15)	13 (12–14)	13 (12–14)	12 (11–13)
Menopausal status	premenopausal	4 (25.0)	31 (25.8)	83 (59.3)	18 (69.2)
	postmenopausal	12 (75.0)	89 (74.2)	57 (40.7)	8 (30.8)
Smoking	current	3 (18.8)	29 (24.2)	16 (11.4)	4 (15.4)
	former	3 (18.8)	16 (13.3)	9 (6.4)	2 (7.7)
	never	9 (56.3)	64 (53.3)	104 (74.3)	19 (73.1)
	missing	1 (6.3)	11 (9.2)	11 (7.9)	1 (3.8)
Hormone replacement therapy	current	1 (6.3)	4 (3.3)	3 (2.1)	1 (3.8)
	former	3 (18.8)	20 (16.7)	14 (10.0)	
	never	12 (75.0)	92 (76.7)	123 (87.9)	25 (96.2)
	missing		4 (3.3)		
Oral contraceptives	current		8 (6.7)	23 (16.4)	5 (19.2)
	former	3 (18.8)	33 (27.5)	37 (26.4)	7 (26.9)
	never	8 (50.0)	49 (40.8)	57 (40.7)	11 (42.3)
	missing	5 (31.3)	30 (25.0)	23 (16.4)	3 (11.5)

^a^Throughout the table BI-RADS breast composition is intended

**Table 2:** Tumor characteristics at diagnosis according to mammographic density at diagnosis

		BI-RADS^a^ a	BI-RADS b	BI-RADS c	BI-RADS d
Estrogen receptor status	positive	5 (31.3)	69 (57.5)	89 (63.6)	20 (76.9)
	negative	11 (68.8)	47 (39.2)	46 (32.9)	6 (23.1)
	missing		4 (3.3)	5 (3.6)	
Progesterone receptor status	positive	4 (25.0)	44 (36.7)	77 (55.0)	15 (57.7)
	negative	12 (75.0)	72 (60.0)	58 (41.4)	11 (42.3)
	missing		4 (3.3)	5 (3.6)	
HER2 status	positive	3 (18.8)	45 (37.5)	38 (27.1)	9 (34.6)
	negative	11 (68.8)	68 (56.7)	95 (67.9)	17 (65.4)
	missing	2 (12.5)	7 (5.8)	7 (5.0)	
Ki67	> 20% (high)	12 (75.0)	89 (74.2)	89 (63.6)	15 (57.7)
	<=20% (low)	2 (12.5)	9 (7.5)	25 (17.9)	3 (11.5)
	missing	2 (12.5)	22 (18.3)	26 (18.6)	8 (30.8)
FNA^b^ axilla	positive FNA	11 (68.8)	80 (66.7)	91 (65.0)	15 (57.7)
	negative FNA		8 (6.7)	10 (7.1)	4 (15.4)
	inconclusive FNA	1 (6.3)		3 (2.1)	1 (3.8)
	no FNA	4 (25.0)	32 (26.7)	35 (25.0)	6 (23.1)
	missing			1 (0.7)	
Tumor size at diagnosis (mm)^c^	median (IQR)	34 (23–40)	30 (21–40)	35 (25–50)	30 (20–40)

^a^Throughout the table BI-RADS breast composition is intended

^b^fine needle aspiration

^c^Measured in mammograms or in ultrasound images. When both methods were conclusive an average measure was used

Patients with pCR following NACT (*N* = 57) were compared to patients without pCR: older, more often postmenopausal, more often with a history of HRT use, less often current oral contraceptive users, more likely to be multiparous (three or more children), and more likely to be smokers or former smokers (Table 3). Age at menarche and first child birth were fairly equally distributed among patients with or without pCR.

**Table 3:** Patient characteristics at diagnosis according to pathological complete response

		pCR^a^	Non-pCR
Number of patients		57	245
Age	median (IQR)	55 (44–65)	53 (44–62)
BMI	median (IQR)	25 (22–28)	25 (23–28)
Number of pregnancies	0	4 (7.0)	34 (13.9)
	1	9 (15.8)	37 (15.1)
	2	16 (28.1)	85 (34.7)
	3+	28 (49.1)	87 (35.5)
	missing		2 (0.8)
Age at first birth	median (IQR)	29 (23–33)	28 (25–33)
Age at menarche	median (IQR)	13 (12–13)	13 (12–14)
Menopausal status	postmenopausal	35 (61.4)	131 (53.5)
	premenopausal	22 (38.6)	114 (46.5)
Smoking	current	11 (19.3)	41 (16.7)
	former	9 (15.8)	21 (8.6)
	never	32 (56.1)	164 (66.9)
	missing	5 (8.8)	19 (7.8)
Hormone replacement therapy	current	4 (7.0)	5 (2.0)
	former	10 (17.5)	27 (11.0)
	never	43 (75.4)	209 (85.3)
	missing		4 (1.6)
Oral contraceptives	current	4 (7.0)	32 (13.1)
	former	15 (26.3)	65 (26.5)
	never	22 (38.6)	103 (42.0)
	missing	16 (28.1)	45 (18.4)

^a^pathological complete response

The distribution of pretreatment tumor characteristics, according to pCR or non-pCR, as presented in Table 4, indicates that patients with smaller tumors, a positive lymph node prior to treatment initiation, high proliferation, negative ER and/or PR status, and/or positive HER2 status were more likely to obtain pCR.

**Table 4:** Tumor characteristics at diagnosis according to pathological complete response

		pCR^a^	Non-pCR
Number of patients		57	245
Estrogen receptor status	positive	18 (31.6)	165 (67.3)
	negative	38 (66.7)	72 (29.4)
	missing	1 (1.8)	8 (3.3)
Progesterone receptor status	positive	12 (21.1)	128 (52.2)
	negative	44 (77.2)	109 (44.5)
	missing	1 (1.8)	8 (3.3)
HER2 status	positive	35 (61.4)	60 (24.5)
	negative	20 (35.1)	171 (69.8)
	missing	2 (3.5)	14 (5.7)
Ki67	> 20% (high)	44 (77.2)	161 (65.7)
	<=20% (low)	3 (5.3)	36 (14.7)
	missing	10 (17.5)	48 (19.6)
Axillary lymph node	positive FNA^b^	41 (71.9)	156 (63.7)
	negative FNA	3 (5.3)	19 (7.8)
	inconclusive FNA		5 (2.0)
	no FNA	13 (22.8)	64 (26.1)
	missing		1 (0.4)
Tumor size at diagnosis (mm)^c^	median (IQR)	28 (20–35)	35 (25–46)

^a^pathological complete response

^b^fine needle aspiration

^c^Measured in mammograms or in ultrasound images. When both methods were conclusive an average measure was used

Tumor biomarker expression differed in some tumors when comparing samples from the pre- and post-neoadjuvant treatment setting. A total of 32 tumors were discordant in PR expression when examining pre- and post-neoadjuvant tumor samples, categorized as PR-positive pre-chemotherapy and after NACT: the remaining tumor cells were considered as PR-negative. A corresponding number of tumors changing from positive receptor status pre-treatment to negative receptor status post-treatment was *N* = 6 for ER and *N* = 11 for HER2, respectively. The number of tumors for the opposite correlation, i.e., change from negative to positive receptor status were *N* = 5, *N* = 9, and *N* = 7 for ER, PR, and HER2, respectively. A total of 80 tumors changed from being highly proliferative (high Ki67-score) to low-level proliferative (low Ki67-score) during NACT and two tumors changed from being less proliferative to highly proliferative during NACT. The characteristics of the remaining tumors in the non-pCR group are listed in Additional file 2.

**Fig. 2:**
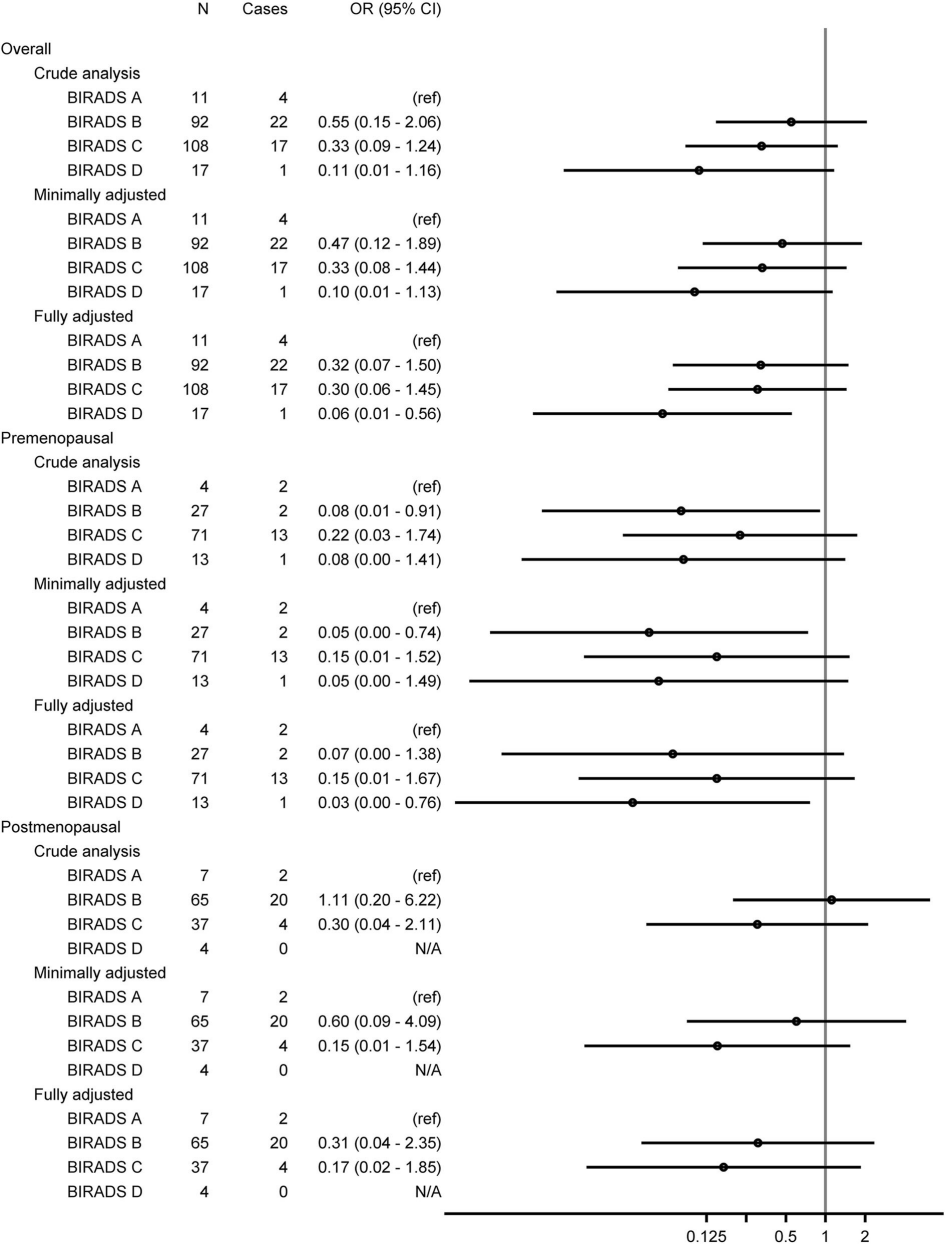
The associations between mammographic density and pathological complete response visualized in a forest plot

A total of 228 patients, of whom 44 (19.3%) accomplished pCR following NACT, had complete data and were included in the logistic regression models. Table 5 shows the association between MD and pCR following NACT in three models, with an increasing number of adjustment variables. In the fully adjusted model, with the most pronounced association of three models, when compared to patients with non-dense breasts (BI-RADS breast composition *a*), patients with more dense breasts had a lower OR of accomplishing pCR on a descending scale: BI-RADS *b* 0.32 (95%CI 0.07–1.50), BI-RADS *c* 0.30 (95%CI 0.06–1.45), and BI-RADS *d* 0.06 (95%CI 0.01–0.56). This association was more pronounced when the premenopausal patients were analysed separately: OR pCR for patients with BI-RADS *b* 0.07 (95%CI 0.00–1.38), OR pCR for patients with BI-RADS *c* 0.15 (95%CI 0.01–1.67), and OR for BI-RADS *d* 0.03 (95%CI 0.00–0.76) (Table 6). The association between MD and pCR following NACT for postmenopausal patients is presented in Additional file 3; the association between MD and pCR following NACT is visualized in a forest plot in Fig. 2.

**Table 5:** Associations between mammographic density at diagnosis and pathological complete response following neoadjuvant chemotherapy - all patients

	B-IRADS^a^	N	N of cases	OR (95% CI)
Model 1	a	11	4	(ref)
	b	92	22	0.55 (0.15–2.06)
	c	108	17	0.33 (0.09–1.24)
	d	17	1	0.11 (0.01–1.16)
Model 2	a	11	4	(ref)
	b	92	22	0.47 (0.12–1.89)
	c	108	17	0.33 (0.08–1.44)
	d	17	1	0.10 (0.01–1.13)
Model 3	a	11	4	(ref)
	b	92	22	0.32 (0.07–1.50)
	c	108	17	0.30 (0.06–1.45)
	d	17	1	0.06 (0.01–0.56)

Odds ratio (OR) for pathological complete response (pCR)

Model 1: crude analysis

Model 2: minimally adjusted (age, BMI, menopause, pregnancies, HRT) analysis

Model 3: fully adjusted (model 2 + ER, PR, Ki67, HER2, and tumor size at diagnosis) analysis

^a^Throughout the table BI-RADS breast composition is intended

**Table 6:** Associations between mammographic density at diagnosis and pathological complete response following neoadjuvant chemotherapy - premenopausal patients

	BI-RADS^a^	N	N of cases	OR (95% CI)
Model 1	a	4	2	(ref)
	b	27	2	0.08 (0.01–0.91)
	c	71	13	0.22 (0.03–1.74)
	d	13	1	0.08 (0.00–1.41)
Model 2	a	4	2	(ref)
	b	27	2	0.05 (0.00–0.74)
	c	71	13	0.15 (0.01–1.52)
	d	13	1	0.05 (0.00–1.49)
Model 3	a	4	2	(ref)
	b	27	2	0.07 (0.00–1.38)
	c	71	13	0.15 (0.01–1.67)
	d	13	1	0.03 (0.00–0.76)

Odds ratio (OR) for pathological complete response (pCR)

Model 1: crude analysis

Model 2: minimally adjusted (age, BMI, pregnancies) analysis

Model 3: fully adjusted (model 2 + ER, PR, Ki67, HER2, and tumor size at diagnosis) analysis

^a^Throughout the table BI-RADS breast composition is intended

## Discussion

This study shows that MD, estimated with the clinically widespread and easily accessible BI-RADS breast composition categorization, can possibly be used as a predictive marker for pCR. Our results show that the likelihood of accomplishing pCR decreases with increasing MD, using BI-RADS *a* as a reference. As shown in the fully adjusted model, the association between MD and pCR was slightly stronger when adjusting for patient and tumor characteristics. This association was more pronounced when the premenopausal women were analysed separately. For postmenopausal patients, the trend was the same for those with BI-RADS breast composition category *b* and *c*, although none of the four patients in the BI-RADS breast composition category *d* achieved pCR; thus, this category was not part of the logistic regression models. Our results are in line with previous studies, claiming that MD holds predictive information for response to anticancer treatment [4, 17, 19]. This study indicates that in the neoadjuvant BC setting, especially for premenopausal women, MD may be considered when making clinical treatment decisions. Our results indicate that premenopausal women with high MD respond poorly to NACT; therefore, they may benefit from other treatment options or at least be monitored closer for treatment efficacy during NACT.

In our logistic regression models, we initially adjusted for hormonal factors known to influence MD, such as age, BMI, menopausal status, HRT, and number of pregnancies [20]. It is known that BC subtypes respond differently to chemotherapy [21–25], and is implied that different tumor characteristics are associated with breast density [26–28], so we subsequently adjusted for tumor characteristics to avoid the potential of confounding from these factors. A biological theory of why tumors in dense breasts respond more poorly to NACT than its counterpart in less dense breasts is, to our knowledge, not presented. BC initiation, progression, and response to treatment are dependent on tumor characteristics, as well as host factors, of which we suggest breast density can be considered to be one. Higher composition of stroma, composed of extracellular matrix proteins, adipocytes, fibroblasts, and immune cells, is considered to contribute to the increased risk of developing BC in mammographically dense breasts compared to less dense breasts [29]. Another theory of a biological explanation in association with breast density and risk for BC is that between breast density and circulating breast mitogens, such as insulin-like growth factor-I and prolactin [30], there is an indication that breast density carries information about the microenvironment in the breast [28]. We hypothesize that the tumor’s surrounding microenvironment is also responsible for different rates of NACT response. Chemotherapy for BC is given as a systemic treatment intravenously, and must traverse vascular endothelium to reach its main breast target [31]. A dense extracellular environment, i.e., high concentration of collagen, hyaluronan and proteoglycans, creates a higher pressure gradient [32, 33] and a higher number of molecules that interact with the drug [34], hypothetically making drug delivery to a tumor in a dense breast more difficult than in a less dense breast. Simultaneously targeting different compartments of a heterogeneous tumor and its surrounding milieu may result in improved patient response and outcomes [35].

MD is a multifactorial dynamic biomarker, changing during a woman’s life, and is a result of genetic [36, 37], hormonal [30, 38], and lifestyle factors [39]. It has been established in the scientific community that MD is a strong risk factor for developing BC, an association that seems to hold true for the BC gene (BRCA)-carriers [40], indicating its great value as a biomarker for risk assessment. MD, as defined, represents the association between the radio-dense (glandular tissue and stroma) and the radio-opaque tissue (fat) [41] – the different components of a breast, which might be a surrogate marker for their interplay in tumor initiation and progression [41]. A less investigated path is MD’s role in terms of response to anticancer treatment, i.e., as a predictive factor for treatment effect explored in this study. A recent study of neoadjuvant treated patients with locally advanced BC showed that patients with low MD at diagnosis have a more favorable disease-free survival rate compared to patients with high MD [19]. A small study of 60 patients with metastatic BC showed better progression-free survival in those with low MD at diagnosis [42]. Our results support the idea that MD holds predictive value and could be considered when deciding on medical treatment for a BC patient.

Pretreatment tumor characteristics split according to pCR status, indicated that patients with smaller tumors, a positive lymph node prior to treatment initiation, high proliferation, negative ER or PR status, or positive HER2 status were more likely to obtain pCR. Except for tumor size, these are all negative prognostic tumor markers [43]. Given that the more aggressive tumors respond better to NACT [44], we can assume that a smaller tumor is more likely to achieve pCR in comparison to its larger counterpart –in line with our study results. Pretreatment tumor characteristics split according to BI-RADS breast composition indicated that tumors in very dense breasts (BI-RADS breast composition *d*) were more likely to be ER-positive, PR-positive, and not highly proliferative (not high Ki67-score) compared to tumors in less dense breasts. Previous studies showed that BC in mammographically denser breasts are often more aggressive in terms of larger tumors and vascular invasion compared to cancers in less dense breasts [27, 29, 45]; the association between ER status [46, 47], PR status, HER2 status, lymph node involvement, mitotic index, and histological grade is incoherent [27, 29, 45]. Hence, regarding pretreatment tumor characteristics in very dense breasts, our findings do not deviate from what one would expect, given the previously described scientific inconsistency.

Different definitions of pCR are used, with two definitions currently considered to be legitimate [48]; one involves the more stringent definition, neither accepting invasive cancer nor cancer in situ in resected breast specimens and all sampled regional lymph nodes: however, the other one accepts cancer in situ in breast and/or lymph nodes. One must know which definition of pCR is used when comparing study results. In terms of patient outcomes, studies show that residual ductal cancer in situ (DCIS) after NACT does not affect local or overall recurrence [49, 50]. Since we use pCR as a surrogate for long-term survival, we include patients with residual DCIS after treatment in the pCR-group, which coincides with the latter of the described definitions [48].

All three sites followed the same clinical guidelines (South Swedish Breast Cancer group) and a group of clinical oncologists alternated between the sites, thus stratification for site was not considered relevant. The local clinical treatment recommendations regarding NACT for BC patients did not fundamentally change during the study’s time span – taxans (96% of the patients received NACT containing taxans) were used since the beginning of neoadjuvant treatment in Southern part of Sweden and none of the included patients received dual HER2-blockage since their treatment preceded this routine; thus, stratification for time was not warranted. A total of 94% of the patients received at least 6 cycles of chemotherapy; reasons for not completing the full 6 cycles were to a great majority related to toxicity and serious side effects.

Some limitations must be discussed. Since all deceased patients were included without consent, the issue of bias according to vital status must be addressed. Since only eight identified patients declined study participation, we estimate this potential bias as minimal. Although high MD it is not stated as an obstacle in guidelines concerning pathological evaluation after NACT for BC [51], a few studies indicate that pathological evaluation is more difficult in a high MD specimen [52, 53]. We expect this potential bias to be negligible in our results. In this study cohort, access to mammograms was restricted to processed images, as raw mammograms were not available. An automated software tool for measuring breast density on raw digital mammograms was thus not possible to use. It is possible that in a few patients with immeasurable tumors due to inflammatory BC, the MD was overrated, since intramammary edema associated with inflammation can increase overall density in the breast [54]. We estimate this to be an insignificant concern, since both cancerous and noncancerous breasts were part of the density assessment and bilateral BC at the time of diagnosis was an exclusion criterion. The overall pCR rate in our cohort is relatively low, likely due to a large proportion of hormone receptor positive BC. The cohort consists of patients receiving NACT as early as 2005 when this treatment regimen was new in Southern Sweden and it is reasonable to speculate that a larger proportion of the patients from the early years was given NACT in order to downsize inoperable tumors, not expecting or aiming for pCR. No published validation studies for software tools that were working on processed images from all vendors in question were accessible when density assessment of this material was made. In future studies, the ambition is to score MD, using a software tool to be compared to the BI-RADS breast composition scoring, as used in this study. We are currently conducting a prospective study in neoadjuvant treated BC patients, in which we analyse raw mammograms with an automated software tool, as well as processed mammograms visually with BI-RADS breast composition categorization (ClinicalTrials.gov Identifier: NCT02306096). The prospective study will investigate the potential of MD as a predictive marker for pCR, and down the line, its relation to prognosis in terms of survival.

A larger dataset is needed in order to increase precision in the results, making the results more generalizable and to better understand the meaning of MD in terms of response to NACT for different subtypes of BC. If our findings are confirmed and lead to clinical implementation of MD as a treatment predictive marker for NACT towards improved BC care, a better understanding of MD as a marker of risk for BC may also be achieved.

## Conclusions

In conclusion, BC patients, particularly those who were premenopausal, with higher MD measured prior to NACT, had a lower likelihood of accomplishing pCR following NACT. If confirmed in future studies, MD may need to be considered in the choice of treatment options for BC patients in the neoadjuvant setting and the predictive role of MD deserves further investigation.

## Supplementary information

**Additional file 1.** Tumor characteristics post-chemotherapy according to mammographic density at diagnosis.

**Additional file 2.** Tumor characteristics post-chemotherapy according to pathological complete response.

**Additional file 3.** Associations between mammographic density (BI-RADS breast composition) at diagnosis and pathological complete response following neoadjuvant chemotherapy - postmenopausal patients.

## Data Availability

The datasets used and/or analysed during the current study are available from the corresponding author on reasonable request.

## Abbreviations

BC: Breast cancer
BI-RADS: Breast Imaging-Reporting and Data System
BMI: Body mass index
CI: Confidence interval
DCIS: Ductal cancer in situ
ER: Estrogen receptor
FISH: Fluorescence in situ hybridization
HER2: Human epidermal growth factor receptor 2
HRT: Hormone replacement therapy
MD: Mammographic density
NACT: Neoadjuvant chemotherapy
OR: Odds ratio
pCR: Pathological complete response
PR: Progesterone receptor
