# Supplementary material for: Mammographic density is a potential predictive marker of pathological response after neoadjuvant chemotherapy in breast cancer
Source: BMC Cancer. 2019 Dec 30;19:1272. doi: 10.1186/s12885-019-6485-4 (PMC6937786; doi:10.1186/s12885-019-6485-4)
Supplement: Supplementary file 1 — Additional file 1. Tumor characteristics post-chemotherapy according to mammographic density at diagnosis. [file 12885_2019_6485_MOESM1_ESM.pdf]

Additional file 1 Tumor characteristics post chemotherapy according to mammographic density at diagnosis

		BI-RADS[a] a	BI-RADS b	BI-RADS c	BI-RADS d
Number of patients		16	120	140	26
Tumor size (mm)	median (IQR)	18 (0 - 30)	8 (0 - 24)	16 (7 - 30)	12 (2 - 19)
Number of positive axillary lymph nodes	0	8 (50.0)	48 (40.0)	61 (43.6)	9 (34.6)
	1-3	2 (12.5)	32 (26.7)	44 (31.4)	8 (30.8)
	4≤	5 (31.3)	36 (30.0)	31 (22.1)	8 (30.8)
	missing	1 (6.3)	4 (3.3)	4 (2.9)	1 (3.8)
Estrogen receptor status	positive	5 (31.3)	50 (41.7)	81 (57.9)	19 (73.1)
	negative	6 (37.5)	29 (24.2)	27 (19.3)	2 (7.7)
	missing	5 (31.3)	41 (34.2)	32 (22.9)	5 (19.2)
Progesterone receptor status	positive	4 (25.0)	33 (27.5)	54 (38.6)	8 (30.8)
	negative	7 (43.8)	47 (39.2)	54 (38.6)	12 (46.2)
	missing	5 (31.3)	40 (33.3)	32 (22.9)	6 (23.1)
HER2 status	positive	1 (6.3)	18 (15.0)	25 (17.9)	3 (11.5)
	negative	10 (62.5)	61 (50.8)	81 (57.9)	17 (65.4)
	missing	5 (31.3)	41 (34.2)	34 (24.3)	6 (23.1)
Ki67	>20% (high)	5 (31.3)	31 (25.8)	28 (20.0)	5 (19.2)
	<=20% (low)	6 (37.5)	46 (38.3)	71 (50.7)	12 (46.2)
	missing	5 (31.3)	43 (35.8)	41 (29.3)	9 (34.6)

a. Throughout the table BI-RADS breast composition is intended
