# Supplementary material for: Mammographic density is a potential predictive marker of pathological response after neoadjuvant chemotherapy in breast cancer
Source: BMC Cancer. 2019 Dec 30;19:1272. doi: 10.1186/s12885-019-6485-4 (PMC6937786; doi:10.1186/s12885-019-6485-4)
Supplement: Supplementary file 2 — Additional file 2. Tumor characteristics post-chemotherapy according to pathological complete response. [file 12885_2019_6485_MOESM2_ESM.pdf]

Additional file 2 Tumor characteristics post chemotherapy according to pathological complete response

		pCR[a]	Non-pCR
Number of patients		57	245
Tumor size (mm)	median (IQR)	0 (0 - 0)	17 (10 - 30)
Number of positive axillary lymph nodes	0	54 (94.7)	72 (29.4)
	1-3		86 (35.1)
	>4		80 (32.7)
	missing	3 (5.3)[b]	7 (2.9)
Estrogen receptor status	positive		155 (63.3)
	negative		64 (26.1)
	missing	57 (100)	26 (10.6)
Progesterone receptor status	positive		99 (40.4)
	negative		120 (49.0)
	missing	57 (100)	26 (10.6)
HER2 status	positive		47 (19.2)
	negative		169 (69.0)
	missing	57 (100)	29 (11.8)
Ki67	>20% (high)		69 (28.2)
	<=20% (low)		135 (55.1)
	missing	57 (100)	41 (16.7)

a. pathological complete response
b. Three patients with pCR in the breast specimen did not undergo axillary surgery.
