# Supplementary material for: Mammographic density is a potential predictive marker of pathological response after neoadjuvant chemotherapy in breast cancer
Source: BMC Cancer. 2019 Dec 30;19:1272. doi: 10.1186/s12885-019-6485-4 (PMC6937786; doi:10.1186/s12885-019-6485-4)
Supplement: Supplementary file 3 — Additional file 3. Associations between mammographic density (BI-RADS breast composition) at diagnosis and pathological complete response following neoadjuvant chemotherapy - postmenopausal patients. [file 12885_2019_6485_MOESM3_ESM.pdf]

Additional file 3 Associations between mammographic density (BI-RADS breast composition) at diagnosis and pathological complete response following neoadjuvant chemotherapy - postmenopausal patients

	BI-RADS[a]	N	N of cases	OR (95% CI)
Model 1	a	7	2	(ref)
	b	65	20	1.11 (0.20 - 6.22)
	c	37	4	0.30 (0.04 - 2.11)
	d	4	0	N/A
Model 2	a	7	2	(ref)
	b	65	20	0.60 (0.09 - 4.09)
	c	37	4	0.15 (0.01 - 1.54)
	d	4	0	N/A
Model 3	a	7	2	(ref)
	b	65	20	0.31 (0.04 - 2.35)
	c	37	4	0.17 (0.02 - 1.85)
	d	4	0	N/A

a. Throughout the table BI-RADS breast composition is intended
Odds ratio (OR) for pathological complete response (pCR)
Model 1: crude analysis
Model 2: minimally adjusted (age, BMI, pregnancies, HRT) analysis
Model 3: fully adjusted (model 2 + ER, PR, HER2, and tumor size at diagnosis) analysis
